# *Gardnerella vaginalis* Enhances *Atopobium vaginae* Viability in an *in vitro* Model

**DOI:** 10.3389/fcimb.2020.00083

**Published:** 2020-03-04

**Authors:** Joana Castro, Aliona S. Rosca, Piet Cools, Mario Vaneechoutte, Nuno Cerca

**Affiliations:** ^1^Laboratory of Research in Biofilms Rosário Oliveira (LIBRO), Centre of Biological Engineering (CEB), Braga, Portugal; ^2^Laboratory Bacteriology Research, Faculty of Medicine and Health Sciences, Ghent University, Ghent, Belgium

**Keywords:** bacterial vaginosis, polymicrobial biofilms, *Gardnerella*, *Atopobium vaginae*, PNA-FISH

## Abstract

Bacterial vaginosis (BV) is the most common vaginal infection among women of reproductive age. A hallmark of BV is the presence of a highly structured polymicrobial biofilm on the vaginal epithelium, presumably initiated by facultative anaerobes of the genus *Gardnerella*, which then becomes a scaffold for other species to adhere to. One of the species often found incorporated in *Gardnerella* mediated biofilms is *Atopobium vaginae*. Interestingly, *A. vaginae* is very rarely found without the presence of *Gardnerella*. However, not much is known regarding the interactions between *A. vaginae* and *Gardnerella* species. This study assessed biological interactions between *Gardnerella vaginalis* and *A. vaginae*. In our *in vitro* model, by using specific *Gardnerella* and *A. vaginae* Peptide Nucleic Acid (PNA)-Fluorescence *In Situ* Hybridization (FISH) probes, we confirmed that *A. vaginae* was able to incorporate a pre-formed *G. vaginalis* biofilm, accounting for up to 20% of the total number of biofilm cells. However, our findings showed that almost 92% of *A. vaginae* cells lost viability after 48 h of mono-species planktonic growth, but were able to maintain viability when co-cultured with *Gardnerella* or after pre-conditioning with cell-free supernatant of *Gardnerella* cultures. While the *in vitro* conditions are very different from the *in vivo* microenvironment, this study contributes to a better understanding of why *A. vaginae* vaginal colonization rarely occurs in the absence of *Gardnerella*. Overall, this highlights the importance of microbial interactions between BV-associated bacteria and demands more studies focused on the polymicrobial bacterial communities found in BV.

## Introduction

Bacterial vaginosis (BV) is the most prevalent bacterial vaginal infection in women of reproductive age (Jung et al., 2017; van de Wijgert and Jespers, 2017; Rosca et al., 2019). BV is characterized by a change in the microbial composition of the vaginal ecosystem where the prevailing *Lactobacillus* spp., associated with an optimal vaginal microbiota, are outnumbered by other microorganisms, including species of the genus *Gardnerella* and *Atopobium vaginae* (Ferris et al., 2004; Verhelst et al., 2004; dos Santos Santiago et al., 2012; Jung et al., 2017; Muzny et al., 2019). Noteworthy, the involvement of *A. vaginae* in BV rarely occurs in the absence of *Gardnerella* (Bradshaw et al., 2006; Hardy et al., 2015, 2016).

It should be also noted that *Gardnerella vaginalis* was the only recognized species in its genus for four decades (Castro et al., 2020), but very recently a whole-genome analysis of 81 *Gardnerella* isolates carried out by Vaneechoutte and coworkers showed the existence of 13 species within the genus *Gardnerella* (Vaneechoutte et al., 2019). Of these 13 species, three new were officially described (*G. leopoldii, G. piotii*, and *G. swidsinskii*) and *G. vaginalis* was amended. Nine genomospecies were defined but not described because the authors did not have the strains (needed for the official description). Following this renewed taxonomy of the genus *Gardnerella*, in this article, the term *Gardnerella* spp. will be used to discuss previous publications, because we cannot rule out the fact that other *Gardnerella* species were involved.

An important discovery of the last decade was the observation that a highly structured polymicrobial biofilm on the vaginal epithelium, presumably initiated by *Gardnerella* spp., becomes a scaffold for other species to adhere to (Swidsinski et al., 2005, 2013). While many bacterial species have been found associated with BV, the contribution of those species to the biofilm formation process is not well documented, remaining unclear its role in the development of BV infection. An interesting example is the case of *A. vaginae*. Evidence of a possible dependent relationship between *Gardnerella* spp. and *A. vaginae* has been demonstrated on BV-associated biofilms (Swidsinski et al., 2005; Hardy et al., 2015, 2016). Nevertheless, as biological interactions in BV-associated biofilms are still poorly understood, we aimed to analyze the interactions between *G. vaginalis* and *A. vaginae*, using a previously described *in vitro* dual-species biofilm model (Castro et al., 2019). We then evaluated cell viability when these bacterial species were grown for 48 h in either mono- or co-cultures through fluorescence *in situ* hybridization (FISH) method.

## Methods

### Reclassification of *Gardnerella* Species

Our collection of fourteen *Gardnerella* spp. isolates first identified by partial sequencing of the16S rRNA coding gene as *G. vaginalis* (Castro et al., 2015) and according to the clade classification system (Ahmed et al., 2012; Castro et al., 2018), were herein reclassified by MALDI-TOF protein profiling as described by Vaneechoutte et al. (2019). Briefly, eight peptide spectra were generated from all strains after ethanol/acetic acid extraction using the Microflex Biotyper™ spectrometer (Bruker Daltonics, Germany). Raw data spectra were imported in BioNumerics Software (Applied Maths, Belgium), used to make one summary spectrum per strain. The summary spectra were then used to classify the strains as *G. vaginalis, G. leopoldii, G. piotii*, or *G. swidsinskii* according to the peptide biomarkers described by Vaneechoutte and coworkers.

### Strains and Culture Conditions

*G. vaginalis* strain ATCC 14018^T^ and *A. vaginae* strain ATCC BAA-55^T^ were used as controls in all the experimental assays. Then, for the bacterial viability experiments, five additional strains of *G. vaginalis*, namely UM121 and UM137 (Castro et al., 2015) and UGent09.01, UGent09.07, and UGent25.49 (Vaneechoutte et al., 2019), and five additional strains of *A. vaginae*, namely BVS065, BVS067, BVS069, FB106b, VMF0907Col23 (Henriques et al., 2012), were used. All strains were grown in supplemented BHI (sBHI) [Brain-heart infusion (Liofilchem, Rosetodegli, Abruzzi, Italy) containing 2% (wt/vol) gelatin (Liofilchem), 0.5% (wt/vol) yeast extract (Liofilchem), and 0.1% (wt/vol) soluble starch (Panreac, Barcelona, Spain)] for 24 h (for biofilm experiments) or 48 h (for bacterial viability experiments) at 37°C under anaerobic conditions [controlled atmosphere composed of 10% carbon dioxide, 10% helium and 80% nitrogen generated by a cylinder (Air Liquid, Algés, Portugal) coupled to an anaerobic incubator (Plas-Labs, Lansing, MI)].

### Biofilm Formation and Biomass Quantification

Dual-species biofilms were initiated by inoculating a 10^7^ CFU/mL bacterial suspension of *G. vaginalis* strain ATCC 14018^T^ into 24-well tissue culture plates (Orange Scientific, Braine L'Alleud, Belgium) and by incubating the plate for 24 h, at 37°C and under anaerobic conditions. After 24 h, planktonic cells were removed, and fresh medium was added to each well. Then, 10^7^ CFU/mL of *A. vaginae* strain ATCC BAA-55^T^ was inoculated in the pre-formed *G. vaginalis* biofilms and incubated for another 24 h. Mono-species biofilms were grown as controls. To quantify the biomass of mono- and dual-species biofilms, we used the crystal violet (CV) method, which is the most frequently employed approach (Peeters et al., 2008; Azeredo et al., 2017). In brief, after the fixation step with 100% (vol/vol) methanol (Thermo Fisher Scientific) for 20 min, biofilms were stained with CV solution at 1% (vol/vol) (Merck, Darmstadt, Germany) for 20 min. Each well was washed twice with phosphate-buffered saline, and bound CV was released with 33% (vol/vol) acetic acid (Thermo Fisher Scientific, Lenexa, KS). To estimate total biomass, the optical density (OD) of the resulting solution was measured at 595 nm. Biofilm assays were repeated three times on separate days, with four technical replicates assessed each time.

### Quantification of Bacterial Populations in Dual-Species Biofilms by PNA-FISH

The bacterial population within the biofilms formed was discriminated according to FISH method (Machado et al., 2013), by using peptide nucleic acid (PNA) probes specific for *G. vaginalis* (Gard162) (Machado et al., 2013) and for *A. vaginae* (AtoITM1) (Hardy et al., 2015). Before counting the percentage of cells detected by PNA-FISH, any non-adherent cells were removed by two gentle washes with PBS and, thereafter, biofilms were scraped vigorously from the well. For mono- and dual-cultures, 30 μL of each bacterial suspension was spread on epoxy-coated microscope glass slides (Thermo Fisher Scientific) and the slides air-dried. Next, cells were fixed, at room temperature, with 100% (vol/vol) methanol, for 20 min, followed by 4% (wt/vol) paraformaldehyde (Thermo Fisher Scientific), for 10 min, and then by 50% (vol/vol) ethanol (Thermo Fisher Scientific) for 10 min. After the fixation step, the samples were covered with 10 μL of each PNA probe and incubated in a hybridization oven (Nahita, drying oven, model 631/2) in humid conditions, at 60°C for 90 min. Afterward, the slides were immersed in a washing solution containing 5 mM Tris base (Thermo Fisher Scientific), 15 mM NaCl (Liofilchem) and 0.1% (vol/vol) Triton X-100 (Fisher Bioreagents, Pennsylvania, USA) for 30 min at 60°C. After this washing step, the slides were air-dried in the dark and at room temperature. Microscopic visualization was performed using filters capable of detecting the PNA Gard162 probe (BP 530-550, FT 570, LP 591 sensitive to the Alexa Fluor 594 molecule attached to the Gard162 probe) and the PNA AtoITM1 probe (BP 470-490, FT500, LP 516 sensitive to the Alexa Fluor 488 molecule attached to the AtoITM1 probe). Twenty fields were randomly acquired in each sample. The number of bacteria was counted using *ImageJ Software* (Rasband, 1997). Biofilm assays were repeated three times on separate days.

### Confocal Laser Scanning Microscopy Analysis of Biofilm Bacterial Distribution

To analyze the bacterial distribution of dual-species biofilms, the intact biofilm structure was evaluated by confocal laser scanning microscopy (CLSM) using the PNA Gard162 and PNA AtoITM1 probes as described above. For this experiment, biofilms were formed as described above but on an eight-well chamber slide (Thermo Fisher Scientific™ Nunc™ Lab-Tek™, Rochester, NY). The CLSM images were acquired with an Olympus™ Fluo View FV1000 confocal laser scanning microscope (Olympus), using a ×10 objective and with a 640 × 640 resolution (pixels). All assays were repeated three times with two technical replicates.

### Coaggregation Assays

To determine the extent of the coaggregation between *G. vaginalis* strain ATCC 14018^T^ and *A. vaginae* strain ATCC BAA-55^T^, we used an experimental model suggested by Reid et al. (1990). Of note, coaggregation assays were carried out using planktonic cultures and aimed to assess the possible mechanism behind the development of bacterial biofilms (Rickard et al., 2003). However, it has been reported that there is not always a direct relation between coaggregation and biofilm formation (Karched et al., 2015). In brief, 500 μL of *G. vaginalis* (10^7^ CFU/mL) was combined with 500 μL of *A. vaginae* (10^7^ CFU/mL) in 24-well plates (Thermo Fisher Scientific) and incubated for 4 h, at 37°C, under anaerobic conditions. The aggregates were visualized using an inverted light microscope Leica DMI 3000B (Leica Microsystems, Wetzlar, Germany) and the score was evaluated as following: 0, no aggregation; 1, small aggregates comprising small visible clusters of bacteria; 2, aggregates comprising larger numbers of bacteria, settling to the center of the well; 3, macroscopically visible clumps comprising larger groups of bacteria which settle at the center of the well; 4, maximum score allocated to describe large clumps, macroscopically visible at the center of the well. Auto-aggregation was assessed for each isolate. All assays were performed in duplicate and repeated three different times.

### Bacterial Viability on Mono- and Co-cultures Under Planktonic Conditions

*G. vaginalis* and *A. vaginae* cell viability was assessed before and after 48 h of planktonic growth in mono- and in co-cultures (contact-dependent interactions), by PNA-FISH, as previously described. In brief, for contact-dependent interactions assays, monocultures were grown as described above (section Strains and Culture Conditions). For dual-cultures planktonic growth, 2 mL of *G. vaginalis* suspension was mixed with 2 mL of *A. vaginae* suspension. As such, each species is present at half of the concentration in the respective dual-culture. Furthermore, the influence of 10 and 50% (vol/vol) of cell-free supernatant (CFS) of *G. vaginalis* on *A. vaginae* cells viability was also evaluated (contact-independent interactions) based on the protocol described by Khan et al. (2019). Briefly, CFS was generated by centrifuging the 48 h inoculum of *G. vaginalis* for 30 min at 3,000 × g. The supernatant was filter-sterilized using 0.22 μm filters and was used on the same day. Filter-sterilized CFS was streaked on Columbia base agar supplemented with 10% defibrinated horse blood (Thermo Fisher Scientific) to confirm sterility. To test the effect of CFS on *A. vaginae*, we added 0.4 or 2 mL of CFS in individual tubes with a *A. vaginae* suspension (final volume of 4 mL) and then the tubes were incubated for 48 h. *A. vaginae* was also grown in control tubes with media containing no CFS. Of note, the effect of CFS of *G. vaginalis* on *G. vaginalis* growth was also analyzed as a control (“self-CFS”). All assays were performed in duplicate and repeated three times.

### Statistics

All numerical data were subjected to statistical analysis using One-way ANOVA test or non-parametric Kruskal-Wallis test, when data that did not follow a normal distribution according to Kolmogorov–Smirnov's test. Statistical software package SPSS 17.0 (SPSS Inc. Chicago, IL) was used. Data are presented as mean ± standard deviation (s.d.), unless stated otherwise.

## Results

### Biofilm Assays

**Figure 1:**
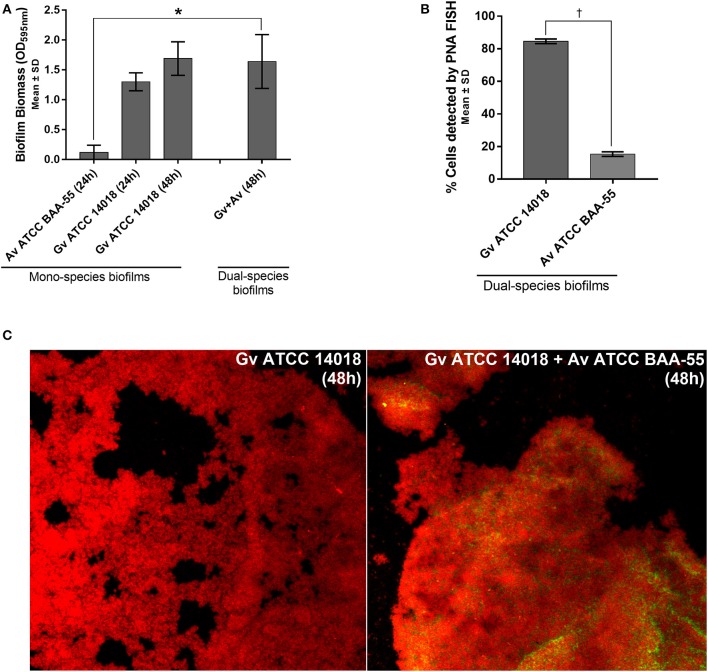
Interactions between *G. vaginalis* strain ATCC 14018 and *A. vaginae* strain ATCC BAA-55 cultured under biofilms conditions. **(A)** Total biomass of mono- and dual-species BV-associated biofilms was determined by staining with crystal violet. Each data point represents the mean ± s.d. of three independent assays, with four technical replicates assessed each time. **(B)** Percentage of cells detected by PNA-FISH for 48 h biofilms. Each data point represents the mean ± s.d. of three independent assays. For each assay, 20 fields were randomly acquired in each sample and the number of bacteria per image was counted using *ImageJ Software*. **(C)** Example of data set on the organization of the dual BV-associated biofilms by confocal laser scanning microscopy (CLSM). *Gardnerella vaginalis* (Gv) and *Atopobium vaginae* (Av) cells were differentiated by hybridization with PNA-probes Gard162 (red color) and AtoITM1 (green color), respectively. *Values are significantly different between the dual-species consortium and the mono-species culture (independent samples *t*-test, *P* < 0.05). ^†^ Values are significantly different between the bacterial populations of *G. vaginalis* and *A. vaginae* in dual-species biofilms (paired samples *t*-test, *P* < 0.05).

We observed that in our *in vitro* conditions, *A. vaginae* was not able to form mono-species biofilms (Figure 1A). Interestingly, although *A. vaginae* did not significantly enhance the dual-species biomass when compared with 48 h *G. vaginalis* mono-species biofilms, this species was able to incorporate the biofilm, accounting for up to ~20% of the total number of cells, as determined by PNA-FISH (Figure 1B). CLSM analysis using specific PNA probes revealed that *A. vaginae* was found well distributed across the biofilm, in small clusters of cells (Figure 1C).

### Coaggregation Assays

**Figure 2:**
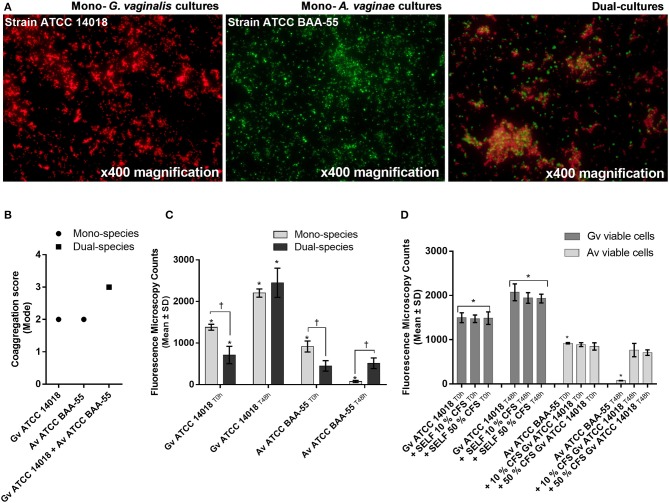
Bacterial interactions in mono- and co-cultures of *G. vaginalis* strain ATCC 14018 and *A. vaginae* strain ATCC BAA-55 cultured under planktonic conditions. **(A)** An example of data set on the organization of microbial aggregates of mono- or dual-bacterial species. **(B)** Coaggregation score of mono- vs dual- bacterial species. Auto-aggregation was also assessed for each bacterial species. Coaggregation score was evaluated as follows: 0, no aggregation; 1, small aggregates comprising small visible clusters of bacteria; 2, aggregates comprising larger numbers of bacteria, settling to the center of the well; 3, macroscopically visible clumps comprising larger groups of bacteria which settle to the center of the well; 4, maximum score allocated to describe a large, macroscopically visible clump in the center of the well. Each data point represents the mode. **(C)** Fluorescence microscopy counts of *G. vaginalis* and *A. vaginae* in mono- and dual-species planktonic cultures. *G. vaginalis* and *A. vaginae* cells were differentiated by hybridization with PNA-probes Gard162 and AtoITM1, respectively. **(D)** Effect of 10% (vol/vol) and 50% (vol/vol) cell-free supernatant (CFS) of *G. vaginalis* on *A. vaginae* viability. The influence of CFS of *G. vaginalis* on *G. vaginalis* growth was also analyzed as a control (“self-CFS”). Each data point represents the average ± s.d. of three experiments. For each assay, 20 fields were randomly acquired in each sample and the number of bacteria per image was counted using *ImageJ Software*. *Values are significantly different between T0h and T48h for each growth condition (Kruskal-Wallis test, *P* < 0.05). ^†^ Values are significantly different between mono- or dual-species cultures for each time point (Kruskal-Wallis test, *P* < 0.05).

Coaggregation-mediated interactions between *G. vaginalis* and *A. vaginae* were also analyzed since co-aggregation is believed to facilitate the integration of new bacterial species into polymicrobial communities (Rickard et al., 2003). As such, we evaluated the ability of each mono- and mixed-species cultures to coaggregate. As shown in Figure 2A, macroscopic clusters formed in the dual-species planktonic cultures contained both species. Furthermore, the presence of both species enhanced the co-aggregation ability (Figure 2B).

### Planktonic Assays

Interestingly, we observed that in planktonic monocultures, ~92% of *A. vaginae* cells had lost their viability after 48 h of growth. However, when growing in mixed cultures, *A. vaginae* was able to maintain the same level of viability, according to the PNA-FISH counts (Figure 2C). Furthermore, by using sBHI medium conditioned by prior growth of *G. vaginalis* to culture *A. vaginae*, we demonstrated that the observed effect was not dependent on physical contact with *G. vaginalis*, suggesting that *A. vaginae* maintains cell viability due to some extracellular molecules produced by *G. vaginalis* (Figure 2D).

**Figure 3:**
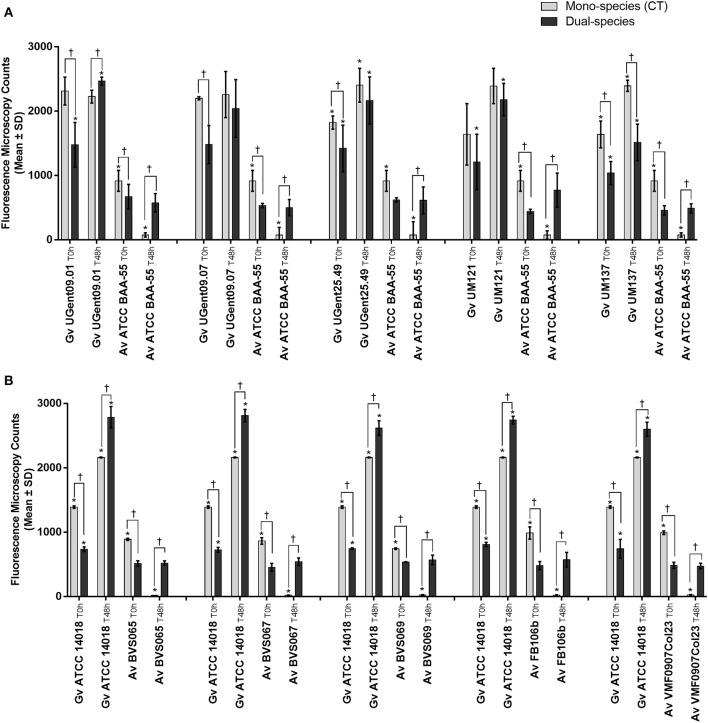
Fluorescence microscopy counts of *G. vaginalis* and *A. vaginae* in mono- and dual-species planktonic cultures. **(A)** Experiments conducted with five other isolates of *G. vaginalis*. **(B)** Experiments conducted with five other isolates of *A. vaginae*. *G. vaginalis* and *A. vaginae* cells were differentiated by hybridization with PNA-probes Gard162 and AtoITM1, respectively. For each assay, 20 fields were randomly acquired in each sample and the number of bacteria per image was counted using *ImageJ Software*. Each data point represents the average ± s.d. of three experiments. *Values are significantly different between T0h and T48h for each growth condition (Kruskal-Wallis test, *P* < 0.05). ^†^ Values are significantly different between mono- or dual-species for each time point (Kruskal-Wallis test, *P* < 0.05).

In order to verify to what extent this was strain-specific, we evaluated *A. vaginae* cells viability in co-culture with five other *G. vaginalis* strains. Due to the recent reclassification of species in the genus *Gardnerella* (Vaneechoutte et al., 2019), we characterized our collection of strains by MALDI-TOF, and selected, for this study, isolates of *G. vaginalis* (Table 1). As shown in Figure 3A, all tested isolates of *G. vaginalis* resulted in a comparable increase in *A. vaginae* cells viability. Identical observations were also observed after repeating the experiment with five other *A. vaginae* strains, as shown in Figure 3B. This confirmed that *A. vaginae* was only viable in the presence of *G. vaginalis* and that this observation is independent of the strains used.

**Table 1:** Reclassification of the *Gardnerella* isolates according to MALDI-TOF protein profiling.

**Strain**	**Accession number^**a**^**	**Clade^**b**^**	**Species identification^**c**^**
*Gardnerella* sp. UM016	KP996686.1	1	*Gardnerella vaginalis*
*Gardnerella* sp. UM034	KP996684.1	4	*Gardnerella leopoldii*
*Gardnerella* sp. UM035	KP996685.1	2	*Gardnerella piotii*
*Gardnerella* sp. UM060	KP996673.1	1	*Gardnerella vaginalis*
*Gardnerella* sp. UM061	KP996674.1	1	*Gardnerella vaginalis*
*Gardnerella* sp. UM067	KP996675.1	2	*Gardnerella* sp.^d^
*Gardnerella* sp. UM085	KP996679.1	1	*Gardnerella vaginalis*
*Gardnerella* sp. UM094	KP996680.1	4	*Gardnerella swidsinskii*
*Gardnerella* sp. UM121	KP996681.1	1	*Gardnerella vaginalis*
*Gardnerella* sp. UM131	KP996676.1	2	*Gardnerella* sp.^d^
*Gardnerella* sp. UM137	KP996682.1	1	*Gardnerella vaginalis*
*Gardnerella* sp. UM224	KP996678.1	4	*Gardnerella leopoldii*
*Gardnerella* sp. UM241	KP996683.1	1	*Gardnerella* sp.^d^
*Gardnerella* sp. UM246	KP996677.1	1	*Gardnerella* sp.^d^

a The partial 16S ribosomal RNA gene sequences of vaginal isolates are downloadable from NCBI. The strains were phenotypically characterized by Castro et al. (2015). UM, University of Minho, Portugal.

b The results regarding the genotyping of Gardnerella isolates based on the clades described by Ahmed et al. (2012) were described in Castro et al. (2018).

c The reclassification of the Gardnerella species was performed by comparing our generated MALDI-TOF spectra with the species-specific peaks defined by Vaneechoutte et al. (2019).

d *MALDI-TOF spectra not matching with any of the described Gardnerella species-specific spectra (i.e., G. vaginalis, G. piotii, G. leopoldii, and G. swidsinskii). Hence, these strains were considered as Gardnerella species (but not G. vaginalis, G. piotii, G. leopoldii, and G. swidsinskii)*.

## Discussion

Despite the evidences suggesting that *Gardnerella* spp. might be the initial colonizer, establishing early biofilm structures to which *A. vaginae* can attach (Swidsinski et al., 2005; Castro et al., 2019; Muzny et al., 2019), there is a lack of studies addressing why *A. vaginae* is almost always accompanied by *Gardnerella* spp. in the vaginal microbiota (Verhelst et al., 2004; Bradshaw et al., 2006; Menard et al., 2010; Hardy et al., 2015, 2016). In this regard, some important attempts have been made to analyze the co-occurrence of these bacterial species in BV-associated biofilms by using the FISH methodology (Frickmann et al., 2017). Importantly, by using a broad range of probes to assess the composition and spatial organization of bacteria in BV-associated biofilms, an *ex vivo* study carried out by Swidsinski and colleagues on vaginal biopsies specimens, demonstrated that adherent biofilms were mainly composed by *Gardnerella* spp. (~60%) and *A. vaginae* (~40%) (Swidsinski et al., 2005). Later, Hardy and colleagues conducted a study on vaginal samples which demonstrated that *A. vaginae* was always part of *Gardnerella* biofilms, but *Gardnerella* biofilms could be found without *A. vaginae* (Hardy et al., 2015). In a subsequent study, Hardy and coworkers demonstrated that adherent *Gardnerella* spp. and *A. vaginae* were visualized, respectively, in 82 and 54% of samples with BV-associated biofilms (Hardy et al., 2016). This was, therefore, the basis for suggesting that *Gardnerella* spp. and *A. vaginae* could establish a relationship in a BV-associated biofilm (Hardy et al., 2016). Furthermore, other studies that addressed the interactions between these two bacterial species, showed that the co-occurrence of *Gardnerella* spp. and *A. vaginae* provides to both bacterial species increased antibiotic resistance (Bradshaw et al., 2006; Swidsinski et al., 2008), and increased expression of genes related to *Gardnerella* virulence (Castro et al., 2019). Such bacterial interactions in the female lower genital tract might have important clinical implications, namely in preterm birth (Menard et al., 2010; Bretelle et al., 2015; Redelinghuys et al., 2017; Mendling et al., 2019). Interestingly, Redelinghuys and colleagues hypothesized that high vaginal concentrations of *Gardnerella* and *A. vaginae* might create a permissive environment for anaerobic Gram-negative bacteria (Redelinghuys et al., 2017). This hypothesis is supported by earlier findings, which showed that women containing high concentrations of *G. vaginalis* and anaerobic Gram-negative bacteria might have higher levels of proinflammatory cytokines and, according to the authors, it could be a reason to the increased risk for spontaneous preterm delivery (Genc et al., 2004; Genc and Onderdonk, 2011).

Given the fact that *A. vaginae* seems to be almost always accompanied by *Gardnerella* in BV biofilms, we hypothesized that *A. vaginae* could be taking advantage of *G. vaginalis* to survive in the vaginal ecosystem. Evidence that supports our hypothesis is the fact that *A. vaginae* does not seem to be able to form mono-species biofilms *in vitro*, as shown by Patterson et al. (2010) and as confirmed herein, at least in the conditions used in both studies. However, it is important to note that the method used in our study to quantify biofilm biomass has some limitations. Despite its widespread use, CV has been associated with lack of reproducibility (Peeters et al., 2008) and absence of a standardized protocol, resulting in a broad variety of staining protocols that make comparison of results between studies difficult (Stepanovic et al., 2007). Additionally, the nonspecific nature of CV does not allow species differentiation in polymicrobial communities (Azeredo et al., 2017).

Interestingly, Faro suggested that a possible explanation for the co-occurrence of *G. vaginalis* and *A. vaginae* could be related to low oxygen levels that prevail in the vaginal environment (Faro, 2006). As such, *Gardnerella* might consume the oxygen, creating a more favorable environment for *A. vaginae*, a strict anaerobe (Faro, 2006). Our results shed new light on these bacterial-interspecies interactions as we demonstrated that the enhanced viability of *A. vaginae* cells was not related to the consumption of oxygen by *G*. *vaginalis* because (*i*) our experimental design involved strictly anaerobic conditions and (*ii*) the physical presence of *G. vaginalis* was not required. It is noteworthy that even contact-independent interactions provided benefits for *A. vaginae*. A similar effect was described before for *Peptostreptococcus anaerobius*, which could only grow in co-culture with *Prevotella bivia* or in medium conditioned by prior growth of *P. bivia* (Pybus and Onderdonk, 1998).

In conclusion, the results from this *in vitro* study demonstrated that *A. vaginae* benefits from *G. vaginalis* to survive, providing a strong indication of the importance of the biological interactions between both taxa. This strengthens the idea that microbial interactions between BV-associated bacteria can be essential in BV pathogenesis. Therefore, future research should address the complex interplay between *G. vaginalis, A. vaginae*, and other BV-associated species. Understanding the molecular basis and biological effect of these inter-bacterial processes may provide novel information fundamental to define new targets and strategies for BV control.

## Data Availability Statement

All datasets generated for this study are included in the article.

## Author Contributions

JC and AR performed the experiments. PC and MV performed the reclassification of *Gardnerella* species. NC and MV designed the study. JC and NC drafted the manuscript. All authors critically reviewed and approved the final manuscript.

### Conflict of Interest

The authors declare that the research was conducted in the absence of any commercial or financial relationships that could be construed as a potential conflict of interest.
